# Opium in Acute Inflammation

**Published:** 1853-04

**Authors:** Edward Cooke


ART. II.—*Opium in Acute Inflammation.* An Inaugural Dissertation, by EDWARD COOKE, M. D. Submitted to the Trustees and Medical Faculty of the University of Louisville, February, 1853.

*History.*—Opium in inflammation, the propriety of which will be considered in the following pages, was introduced to the notice of the profession by the late Dr. Armstrong. He, however, regarded it rather as an useful adjuvant than as a specific—combining with it calomel, and other articles of the Materia Medica, supposed to assert a more absolute and well-defined influence over the morbid action. Some pains have been taken, and considerable research made, with a view to ascertain, if possible, who was the first person that gave to the public the evidences of the utility of opium alone in the treatment of inflammation. The result has been highly flattering to one of our own distinguished countrymen. Dr. Christison, as early as 1841, issued a treatise "On Opium in Acute Internal Inflammation," which was published in the February No. of the Edinburg Monthly Journal for that year. But in several of the diseases in which he recommended its use—in all those of much severity—he invariably premised the abstraction of blood, regarding it as one of the essential elements of success. Previous to this time, however, an American physician—Dr. Barbour—published, in the Am. Journal of the Medical Sciences, an account of his practice in inflammation, consisting in the withdrawal of immense quantities of blood, and the immediate administration of large doses of opium. His paper is dated August, 1840. Dr. Marshall Hall, however, had adopted this mode of practice several years previously. Dr. Stokes has related several cases of peritonitis from perforation, treated by Dr. Graves, with opium alone, but as Dr. Graves, in his publications subsequent to the treatment of these cases, does not rely upon that practice in inflammation generally — not even in peritonitis — and as the opium, in the recorded cases was administered because the circumstances attending them precluded the use of the lancet, calomel, etc., we cannot, in justice, assign to him the honor which more properly belongs to another.

I presume, then, to claim for Dr. Alonzo Clark, of New York, whatever merit may attach to the authorship of this practice; for, as will be shown in the article on peritonitis, he trusts in the treatment of that disease to opium alone.

*Pathology of Inflammation.*—Before entering upon the subject proper of this essay, it is desirable that we should entertain correct notions of the *pathology* of inflammation; but as the space within which custom confines an inaugural dissertation is too limited to allow a thorough investigation of any subject, particularly of inflammation, I shall have to restrict my remarks to generalities, descending to particulars only when the obscurity of abstraction may so demand. I will, therefore, omit a discussion of the various theories propounded since the time of Hunter; presenting, in a form as concise and condensed as the nature and importance of the subject will admit, in addition to my own views, a summary of the theories promulgated by the most distinguished writers of the past and present. In the theories of Hunter, Cullen, Henlé, Wilson Philip, and of others, the capillaries are regarded as the essential seat of the inflammatory process; its phenomena being ascribed to some particular morbid action or condition of those vessels, to debility, spasm, obstruction, increased action, etc. It is the opinion of Mr. Travers that *all* the tissues are implicated; of Liebig, that the diseased tissues undergo an unnaturally rapid oxydation; whilst a few, Dr. Dewitt for one, consider the organic cell to be the main element involved. These various theories, if such they may be called, relate more or less to mere local action, the explanation of the phenomena being grounded upon suppositions which afford us most ingenious specimens of that style of argumentation called by logicians and rhetoricians *petitio principii.* From no one of these, nor from all combined, have we derived any information of practical utility—nothing, that is, in addition to what was previously known.

When we consider that inflammation has its commencement in irritation, that irritation results from some unnatural or immoderate impression made upon the nerves, *that there can be no inflammation without previous irritation, and that irritation presupposes a nervous system,* we have possessed ourselves of a most important fact—a fact which alone can form a sure "stand-point" for reasoning on inflammation. Again, when, by the discoveries of Marshall Hall, we find that all the sphincters and muscles of the body are regulated and presided over by nerves, that almost every organ in the frame-work of man is known to have "a moving and directing power," it is impossible to believe that so important a system as the arterial should be without such a controlling influence. And, in truth, Wilson Philip, and Flourens have demonstrated, by carefully devised experiments, and Sir C. Bell has declared his opinion to be, that the smallest arterial twig is supplied with its nervous filaments, these filaments coming from the system of organic life. If the ganglionic system be subservient to the purposes of life—if all the functions of the human economy be dependent on it for a supply of its vivifying influence—if life may be sustained by an animal deprived of the contres of the other systems of nerves, this being preserved intact—surely we may safely infer that the arteries, agents so essential to the maintenance of existence, receive their share in the distribution of the filaments sent out from the ganglionic system. In corroboration of such an inference on my part, the positive evidence of many anatomists and physiologists might be adduced. The acephalous and anencephalous monstrosities of Lawrence and Hall, it seems to me, should be sufficient to determine this question. They prove not only that existence is maintained through the influence of the ganglionic system, but that originality and growth of the tissues are dependent on it. Witness the action of chloroform, life being preserved, according to Dr. Hall, so long as the ganglionic centres remain unaffected by it.

Consciousness may be suspended, the powers of motion be withdrawn, yet the patient lives, the heart continues to beat, respiration goes on, though slowly and imperfectly, and, after a variable period of time, the patient is restored to his consciousness, and recovers the faculty of motion. A distinguished living physiologist, Dr. A. Clark, to whose lectures the author has often listened with pleasure and admiration, says, in enumerating the functions of the ganglionic centres, they *control* the blood-vessels, to which their filaments are most plentifully distributed; and, if the sympathetic filament going to the eye be cut, that organ becomes immediately suffused with tears. The views of M. Remak concerning the physiology of the ganglionic nervous system are very plausible and entertaining, even though they be not founded in fact. It is his opinion that the animal economy possesses two sensories, the cerebro-spinal axis, and the great ganglia of the sympathetic. He says there are some persons, he himself being one, who have the power of calling into increased action the peristaltic movements of the intestines without the aid of the abdcminal parietes. He therefore assigns to the ganglionic centres the function of volition. As "in the cerebro-spinal system of nerves two orders of action take place — the perception of sensation, and the reactions of volition" — so "two analogous actions take place in organic life — organic perception, or, as it is called, hallerian irritability, and reaction, or the function of organic reflection." Dr. Davey regards the solar plexus as the centre of this system, also as the nisus formativus of the embryonic germ. However this may be, it proves that distinguished physiologists are becoming more and more dissatisfied with the present received opinions of the functions of the nervous system; and, when the popular theories of to-day are overthrown by experiments more ingeniously devised, and more thoroughly conducted, our *pathological* basis will become more sure and steadfast, and the principles of treatment will assume a fixedness which mere speculation will be unable to subvert. Physiology is daily adding to our stock of pathological knowledge, whilst scientific investigations and logical induction are leading to more correct notions of physiological action. These agencies, united to a cautious and rational empiricism in the department of therapeutics, if working to a successful issue, will place the *science* of medicine, the boast of the last hundred years, beyond the envious cavillings and reproaches of her selfish adversaries.

The foregoing principles, together with the experiments and reasoning of J. G. Davey, have led me to the opinion that the ganglionic system is the essential to the continuance of life; that its branches and filaments are distributed to the blood-vessels of the body; that it necessarily presides over the functions of organic life, and bears an important relation to those of animal life; that the least derangement of these nerves gives rise to morbid action in the parts to which they are distributed; that the causes of the disturbance being various necessarily engender varieties of morbid action, cold and moisture producing inflammation, malaria periodic fevers, and animal effluvia fevers of a more permanent type, etc.

My opinion, then, summarily expressed, of the pathology of inflammation is, that irritation of the organic nerves is the "*point de départ;*" the influence or result of the irritation being propagated to the blood-vessels through the medium of the numberlesss threads which these nerves send out to all parts of the body. Be it asked, upon what principles I accouut for the changes which ultimately take place in the circulating current, the viscera, etc., I shall reply by demanding an explanation of the laws of electricity and galvanism, or of the operations of mercury and strychnia. Why certain emotions produce a flow of tears, or mantle the cheek with the blush of modesty; why emotions of a different, or even of a like, kind cause an evacuation of the bladder, or an erection of the penis, are as easy of solution as the hidden and inexplicable causes of various morbid states of a nervous character.

*Pathological Inferences.*—"If debility of its fine threads (the sympathetic) be a sufficient cause to throw into dissonant action the heart and the arteries in some cases, should we not be inclined to abandon the practice which rests on depletion, counter-irritation, and treatment of this kind, and substitute in their places remedies and a regimen which may fortify and sooth the nerve?" If dropsy, for instance, be caused by an obstruction in the portal system, and if this obstruction be owing to debility dependent on an atonic condition of the nervous system, would it not be unscientific to harass and exhaust our patients with hydragogue cathartics and wasting diuretics? Or should we, as is our aim in neuralgia, endeavor to restore to the nerves their accustomed tone, and again bring into healthful action their disordered functions? Some few have contended that the tone of a nerve once impaired could not be restored. Such have not benefitted by the actions of strychnia in lead-paralysis, and of the carbonates of iron and potash in neuralgia. This view of the supposed disturbed functions of the sympathetic is strengthened, somewhat, by the analogous actions of strychnia and the galvanic current upon the disorders of certain organs exclusively under the control of this sytsem, both as regards the kind of power acting upon the nerves, and its influence upon this particular system of nerves. If, then, *inflammation* be caused by influences acting primarily upon the nerves, irritation being the connecting link, should the employment of remedies calculated to soothe the nerves and quiet the irritation, be considered empirical?

The essential cause of intermitting fever is said to be miasmatic; and this aërial-like poison is supposed by many to operate upon the system at large, through the agency of the nervous system. Now, according to the experiences of Drs. Lind and Trotter, a paroxysm of intermittent may be either cut short or effectually prevented by large doses of opium. In case of failure, quinine, or some other nervine, will most probably effect the cure. These articles produce remote effects, operating primarily upon the nerves; through them, secondarily, upon the fluids and solids of the body.

Intermitting fever, however, is not an inflammation; nor do I contend that the causes of the two are identical in their nature. Neither should we be correct in viewing the so-called continued fevers as inflammatory, nor in assigning to them, and to intermitting, an identity of cause and nature. But *I do contend*, whatever be the peculiar and characteristic differences of cause, whether koinomisasmatic, giving rise to periodic fevers; whether idiomiasmatic, engendering fever of the continued type; or whether consisting in moisture and diminished temperature of the atmosphere, fruitful sources of inflammation; I do contend that all these agents of disease produce their primary effects upon the nervous system; that the varying symptoms are assignable to peculiarities of cause, which peculiarities are not so well marked, nor so well understood, as is perhaps imagined; that the *principles* of treatment which guide us in the management of one, furnish us with remedies best adapted to the cure of the others; the treatment being in all cases modified by a knowledge of the causes, and by other circumstances which may arise in consequence of age, idiosyncrasy, habits, condition of system, etc., predisposing to excessive activity and violence, or to debility and prostration. The case-books of many practitioners, who have written to convey information, rather than to uphold long-cherished theories, furnish us with facts which shed a gleam of light upon the results which would attend a practice based upon the principles herein set forth. Drs. Lind and Trotter have left us unmistakable evidence of the value of opium in periodic fevers; during the past summer many members of the profession were furnished an opportunity of manifesting their incredulity by an account of the extraordinary success which Dr. Henry, of Illinois, had obtained by the administration of large doses of opium in continued fever, and I sincerely trust that the following pages may carry conviction to the minds of many, of its great efficacy in inflammation. The author is acquainted with a young practitioner, of this State, an alumnus of this University, who, ever since he began the practice of his profession, has been in the habit of treating both periodic and continued fevers with opium and quinine, sometimes by one alone; most frequently by a combination or alternation of the two. He assured me of the

*almost invariable* success with which he had met. Give me, said he, opium and quinine, and I can do more against the fatality of these fevers than I have ever known accomplished by other remedies. My talented preceptor, with whose *practice* in continued fever, however, I am not conversant, has taught me what I know of its pathology; and no physician has been more successful in its treatment than he.

The similarity in the operation of the causes of these fevers—the continued and periodic—is shadowed forth in the course they pursue. So like, indeed, are the phenomena of remitting and typhoid fever, that some physicians, of whom Prof. Flint, I believe, is one, consider the cause of the former in such cases to be of a mixed nature, partaking of that which produces what, in the language of such a theory, might be called ordinary remittent, and of the nature of that which gives origin to continued fever. Notwithstanding the great respect and admiration I entertain for the talents, the learning, and the philosophically acquired experience, of Prof. Flint, I shall take the liberty, on this occasion, to differ from him, though the limited space allotted myself will prevent my specifying reasons for so doing. But to resume; remitting fever differs essentially from intermitting *only* in the *intensity* of the operation of the cause. Remitting, especially if it is to prove fatal, not unfrequently assumes the type of a continued fever; indeed, the last stages or periods of remitting are more akin to continued fever than to intermitting. Remitting, as it is assuming the continued form, just at that period in its progress which may be called the transition stage, may very easily be mistaken for typhoid fever. In a word, intermitting runs into remitting; remissions become intermissions; remitting takes on the garb of continued; patients afflicted with the latter sometimes have such well-marked exacerbations and remissions as to lead a physician to a most doubtful diagnosis; the diseases shift about, exchanging and interchanging symptoms, if I may so speak, to such a degree, now presenting one type, anon another, as scarcely to leave a doubt that the treatment proper for one might be righteously enforced in the other forms; provided, always, we properly consider the modifications pointed out in a preceding paragraph.

In a large proportion of those patients who die of one or the other of these forms of fever, in almost all, autopsies will reveal inflammatory lesions in some one or more of the viscera. These lesions are not *essential* to fever—nor is any single symptom of fever or inflammation *essential*, as they may and do exist without some one of the symptoms which ordinarily pertain to them—but the frequent association of the fevers and lesions points to some connection between the febrile and inflammatory processes; which connection, though ill defined and much less appreciated, and, to all *appearances*, wanting in some rare instances, may nevertheless exist in all, and, if more thoroughly investigated, might lead to the happiest practical results.

I would not have any one infer that I deny to the humyral pathologists the partial correctness of their views of fever. It *is* a blood disease; but it is something more. It first affects the nervous system; at least the symptoms which first lead us to suspect fever, are of a like nature with those we observe in nervous diseases generally. It is only during this stage of the operation of the cause, the period of access or formative stage, that opium can be relied on, to the exclusion of other remedies not strictly hygienic. After the disease has become *fully* developed, the blood changed in its qualities, and, through it, intercurrent changes of the solids have been induced, we must not, we cannot, with propriety, confine ourselves to opium alone. In the early stage, no agent is more useful, none has been found more successful. Prof. Flint having perused the paper of Dr. Henry, thought fit to give it a trial. It was accordingly administered to five typhoid patients, but not in so large doses as were recommended by Dr. Henry. For this I cannot account, since Dr. H. so strongly insists that it should be given in large doses — from four to six grains. Notwithstanding the smallness of the quantity given, it proved successful in two cases, in one was of considerable benefit, whilst in the other two it completely failed. Had the remedy been given in larger doses, might not all the patients have recovered? Dr. Henry, doubtless, would respond in the affirmative. And might not the disease in the two last cases have been of longer standing than the two first? Prof. Flint, if I remember correctly, did not state in his lecture.

As I am wandering from the more immediate object of this essay, I must hasten on, leaving this part of my subject, it must be acknowledged, very imperfect and incomplete. It is an interesting topic, worthy the consideration of stronger minds, to whom I hesitatingly submit the foregoing remarks, in the hope that, if worthy, they may engage their attention; if unworthy, may be excused as emanations from a fancy which will be corrected in its wandering by a silent criticism.

*Physiological Effects of Opium.* — The diversity of opinion which has long existed, and still exists, respecting the physiological and therapeutical effects of opium, renders it exceedingly difficult for a student, trusting to his books for information, to form an opinion even *approximating* to satisfaction. I shall therefore speak of its effects as coming within the range of my own experience, and within the range of the experience of those whom I believe to have most carefully and impartially investigated the subject.

The physiological effects differ according to the quantity administered. In small doses, opium accelerates the pulse and increases the heat of the surface, produces fulness, or a *sense* of fulness, of the head, numbness, partial insensibility to external impressions, indisposition to bodily exertion, with a vague unconsciousness of, or indifference to, the scenes occurring around: and after sleep, which continues for a period varying from four to eight or nine hours, the person awakes with a sense of oppression of the brain, with thirst and dryness of the fauces, and with listlessness of both mind and body. The secretions from the mucous surfaces are all this time checked, the cutaneous transpiration increased, the renal diminished, whilst the hepatic probably remains unaffected.

By still larger doses no excitation is produced; if there be, however, it is very transitory, sedation almost immediately succeeding. According to Dr. Christison (Dispensatory, p. 681), the effects of a full medicinal dose are, in part, "bluntness of the external senses, and obscurity of the faculties of the mind, gradually passing to profound stupor; and it is rare that exaltation of the mental powers, or increased acuteness of the external senses, precedes these effects." The number of respirations is reduced, the pupils usually contracted, the muscles relaxed, the action of the heart and arteries diminished, and the whole cutaneous surface not unfrequently bathed in perspiration, though usually only covered with moisture; the last effect being, according to my own experience, invariably followed by a diminution in the frequency of the heart's contractions.

It is desirable, but it would not, perhaps, be expedient, to analyse more carefully the effects of opium upon the different systems and organs of the body. A few observations must suffice.

*On the nervous and vascular systems* it is excitant in small doses; in full medicinal doses, it is sedative or depressant.

*On the muscular tissue* it evidently produces relaxation, as is manifested by the power it exercises over the uterus and bladder in arresting the pains of labor, and checking the excretion of urine; over this tissue generally, by allaying spasm and quieting the pains of contraction.

*On the mucus tissue*, it diminishes sensibility, and changes the action of the secreting vessels; lessening and inspissating their secretions.

*On the cutaneous system*, sensibility is blunted, excretion powerfully increased. "In a fatal case which I examined judicially," says Dr. Christison, "the sheets were completely soaked to a considerable distance around the body" (Persira). The degree of action upon sensibility and the skin will be in direct ratio to the quantity administered, up to a certain point. The difference in effect upon the mucous and cutaneous surfaces is, perhaps, to be referred to that close intimacy of structure and functional sympathy characteristic of the two tissues.

*On the glandular osgans:* The experience of the profession is not sufficiently ample or consistent to justify the assertion that opium checks or increases the secretions of these organs. In diabetes, the quantity of urine excreted is certainly diminished by opium, but whether this is owing to a direct action upon the kidneys, or to paralysis of the muscular coat of the bladder, or that close sympathetic relation which is known to subsist between these organs and the external integuments, has never been demonstrated. To stimulate the energy of the cutaneous function is one of the main palliative indications in the treatment of this disease. My own opinion, notwithstanding the weighty authority of Dr. Christison to the contrary, is that the renal secretion is diminished by opium, the reason last assigned being sufficient, in my humble estimation, to account for this effect. Says Dr. Beck, in his Materia Medica, "I have known it absolutely arrested for eighteen hours by an opium suppository."

It is said that the the biliary secretions are also checked, because of the pale color of the feces; yet Sproegel (*vide* Persira) found "the choledic ducts of animals, to whom opium had been given, filled with bile." Dr. Christison thinks the hepatic secretions are unchanged by opium, or "inspissated merely," in consequence of its discharge being impeded. Whether this be true or untrue, I am unable from my own experience to say.

*Application of Opium in Acute Inflammations.*—I proceed, in the next place, to discuss the propriety of opium in various inflammations, which I propose to classify accordingly as they affect (1) the mucous, (2) the fibrous, and (3) the serous tissues.

1. *Inflammation of Mucous Membranes.*—Perhaps no remedy at all applicable to the treatment of inflammation attacking the *air passages* has met with so little favor from the profession as opium, which is mainly due, I conceive, to erroneous notions and groundless apprehensions of the action of the proposed remedy, and to mistaken views of the indications presented by the various inflammatory affections of this track. It has already been remarked that secretion, in common with the functions of organic life generally, is presided over and regulated by the ganglionic centres; that a certain normal condition of these centres is essential to the maintenance and proper regulation of the secretions; that any cause operating to the disturbance of the former, interfere with the latter; and, *e converso*, disturbance in the balance of the secretions is an evidence of nervous derangement. Now, of all the functions rendered irregular by the inflammatory and febrile processes, no one is so constantly affected as that of the secreting vessels, and this effect is the main source of danger in many of the inflammations, as laryngitis, croup, inflammatory diseases of the encephalic membranes, etc. To prevent abnormal secretion then, or, in other words, to prevent effusion, becomes undoubtedly the main object in the treatment of such diseases. Especially is this true of those terrible maladies which are so rapid in their course, and which prove so fatal in their terminations—croup and laryngitis, and those diseases of the brain in which the most unfavorable events seem to be owing entirely to effusion. Every one will admit that if we could prevent, in laryngitis, the oedema of the glottis consequent on serous infiltration of the sub-mucous areolar tissue, we would, with tolerable certainty, thereby rescue our patient from almost inevitable death. How, and upon what principles, should we manage a case of dysentery or inflammatory diarrhea? What remedy, above all other remedies, has been found most successful in fluxes from the gastro-intestinal mucous membrane? Are the anatomical and physiological differences between the gastro-intestinal and the gastro-pulmonary mucous surfaces so great, are the symptoms given out by inflammations of the two portions so widely at variance, as to demand an observance of one set of principles in the treatment of the former, and of a contrary or widely different set in the management of the latter? Or, the principles being the same, are remedies appropriate in the one case, improper in the latter?

Besides, the morbid appearances are similar, identical post-mortem lesions being observed in an inflamed trachea that meet our eyes when inspecting an inflamed duodenum. Then what evidence can there be, what reason, that opium is hurtful in inflammation of the air passages? Not experience surely, for they who testify against it have never tried it. I wish here to correct a very erroneous notion entertained by almost all writers in regard to the effects of opium in inflammation. Say they, opium checks secretion, and therefore must do harm. It is a curious *fact*, that in the early stage of inflammation, during the first twenty-four or seventy-two hours of ordinary cases before secretion has taken place, if opium be given in large doses secretion will be prevented. If, however, secretion has taken place, or is about to take place, opium, by relieving the morbid action going on in the part, will act as an expectorant. Indeed, it is the best expectorant that could be given in such cases.

That the *former* assertion is true will be proven by the cases which I shall take the liberty to quote from an article by Dr. Christison, referred to on a previous page; that the latter also is no less true I have the warrant of Prof. Flint, Who can gainsay the authority of two such men? an authority founded in experience, and by daily experience confirmed and strengthened.

*Coryza, Catarrh and Influenza.*—One day, early in my medical career, whilst reading the dry plan of cure recommended by Dr. C. B. J. Williams — which, by the way, is more intolerable than the diseases themselves — I conceived the opinion that *opium* might exert some influence over those affections, as also over others of the same tissue, the tendency of both plans being to inspissate the secretions from the inflamed surfaces. Dr. Williams, it will be remembered, insists that the patient must abstain from the use of drinks so soon as his *cold* comes upon him, or within a short period thereafter. Dr. Watson observes, "if you practice the old maxim which says, '*venienti occurrite morbo*,' you may occasionally stop a cold on the threshold, as it were, by an opiate." He then relates a case in which this remedy, having been tried by accident, operated like a charm, and alludes to several others in which it maintained its virtues. Although coryza and ordinary catarrh are not, strictly speaking, of an inflammatory nature, I shall nevertheless speak of them after the manner of our nosologists; and it may, practically, be well enough to do so, as catarrh without fever demands as much consideration, and is often attended by symptoms as urgent, as some purely inflammatory affection about the fauces, etc.

For the following extracts, the author is indebted to an admirable paper by Dr. Christison (already referred to) entitled, "The use of Opium in Acute Internal Inflammation." I cannot do better than to quote his language. "Of such diseases there are at least four which may often be thus successfully treated without almost any other remedy, viz: coryza, catarrh, influenza, and dysentery." "For *twenty years* I have been accustomed to see it (coryza) stopped at once by a full opiate given on the first day of its appearance. Let the patient avoid food after dinner, use liquid sparingly, take a full dose of muriate of morphia, or Bartley's solution, at bed time, and breakfast before getting up next morning, and he will then commonly find the secretion from the nostrils permanently inspissated, and the complaint either gone entirely, or, at any rate, no longer a source of particular annoyance." His treatment of catarrh, either with or without fever, and of the influenza, consists in much the same mode of procedure, though in febrile or inflammatory catarrh, he usually substitutes for morphia or Bartley's solution, the Dover's powder. The treatment should not be delayed, if possible, beyond forty-eight hours from the commencement of the disorder.

*Acute Tonsilitis*—I again avail myself of Dr. Christison's admirable paper. "When I saw her, on the fourth day of the disease, the tonsils, greatly enlarged, especially on the right side, blocked up nearly the whole throat. * * * * * A large blister had been applied to the throat, and purgatives taken freely, but without the slightest benefit. The attack seemed too far advanced to be averted. But as fluctuation could not be detected in the larger tonsil, Dover's powder was ordered as in the former case (10 grains every half hour) Sweating ensued; ere long warm drink could be swallowed with no great difficulty; and when the perspiration had thus been kept up for twenty-four hours, the pulse had fallen to 64 (from 116), and the patient entirely recovered." She had previously been subject to attacks of this kind, and never, until the above plan of cure had been enforced, had she enjoyed any immunity from the disease. "This lady has never had another attack, though five years have elapsed since the one now described." Dr. Christison thinks sweating essential; it should be kept up for some time. The sweating, however, is only an indication that the opium has produced its full effects; and the continued perspiration is evidence only of the continued action of the remedy.

*Pneumonia.*—Considering pneumonia to be an inflammation of the lining membrane of the air vessels, which partakes more of the character pertaining to the mucous than to any other tissue, I might, with propriety, speak of the power of opium in its cure, but time, and the space already occupied bid me to hasten on. It may be reremarked, however, that Dr. Christison, and other practitioners of eminence, have found it, preceded by bloodletting, to exert a positively sanative control over this disease. In my book of notes from the lectures of Prof. Flint, I find opium very highly recommended. Prof. F. does not confine its influence to the mere *assuaging* of *pain*, but asserts, with Dr. Christison and others, that it exercises some influence, direct and positive, over the inflammation itself. Some German physician, whose name I cannot now recall, has successfully treated many cases of pneumonia by chloroform. I mention this fact, derived from the lectures of Prof. Rogers, to add strength to the view which I have attempted to give of the pathology of inflammation. Since writing those portions of this essay which treat of catarrh, coryza, etc., and that in which I briefly alluded to croup and laryngitis, I have had the pleasure of hearing the principles which should guide us in the treatment of those affections confirmed by Prof. Flint. I was not aware that any one had ever exercised the boldness to propose, much less to practice, the administration of opium in true membranous croup. I shall speak nothing more of these diseases, nor would any thing from my pen add to the virtues of a remedy pronounced good by an author so well known, and a lecturer so eminently popular, as Prof. Austin Flint.

*Dysentery.*—The testimony of the profession to the happy effects of opium in this species of inflammation is so harmonious as to render only a few words necessary. "Within the last few years I have prescribed large doses of opium, after blood-letting, in at least a hundred cases of acute and sub-acute inflammations of the abdomen, and always with obvious advantage" (Armstrong, art. Dysentery). "Practitioners are too much afraid of opium in inflammations, especially of the abdominal viscera." * * * * "The *quantity* of opium should never be considered in such cases, but only the *effect.* It must be given so as to subdue pain and irritation, whatever be the magnitude of the dose." (Dr.Johnson). "Were the same cases again placed under my care [cases he had met with during the epidemic in Dublin, in 1818], I would not hesitate to give opium in doses of four or five grains, as it was the opium chiefly which seemed to me to arrest the progress of the inflammation." (Stokes, art. Dysentery). The opium had been given in combination with calomel, preceded by venesection. "In severe epidemic dysentery, as it occurs in this country, the cure, when early begun, may be commonly trusted, if I may rely on my own experience, to opium, not used, however, 'with caution,' but given boldly and often." (Christison, in journal referred to on page 281). 'With caution' refers to what Dr. Persira says of the ill-effects of large doses of opium in dysentery. In his lecture on dysentery, Prof. Flint insisted that opium might be given during the 24

hours in quantities ranging from five to fifty grains, provided the symptoms were of so urgent a character as to demand heroic treatment. He related a very interesting circumstance bearing directly upon that portion of this essay in which I have spoken of the connexion existing between fever and inflammation. There were in a certain alms-house—I suppose, in Buffalo—a great many persons crowded together; the rooms were poorly ventilated; during one season dysentery prevailed among the inmates, whilst in another season they were scourged with some form of continued fever. It is the opinion of Prof. F. that the specific cause in both cases was doubtless the concentration of human effluvia. I shall not comment on this singular coïncidence, it speaks for itself.

2. *Inflammation of Fibrous Tissue.*—*Rheumatism* is essentially an inflammation of the fibrous tissue, yet we should err in considering it a mere local affection. Metastasis from the joints to internal organs is by no means rare, pericarditis or endocarditis being usually the result. It is practically of no importance to determine whether the joints or cardiac membranes be primarily affected, for the disease, being constitutional, may attack one or the other alternately, or both at the same time; and the only difference in the treatment of the two arises from the greater violence in the symptoms in the heart disease, and from the paramount importance of the organ inflamed. Colchicum has been, or was, the most lauded internal remedy for rheumatism, but I think it more appropriate to the gouty than to the acute articular form. Opium was introduced to the notice of the profession by Dr. Hope, and of its efficacy he was informed, so says Dr. Watson, by Dr. Chambers. The opinion entertained of its value, by that distinguished writer, has been concurred in by Drs. Graves, Stokes, Corrigan, and a host of others on both sides of the Atlahtic.

In the management of acute rheumatism, Dr. Christison orders, after the pretty copious abstraction of blood, ten grains of Dover's powder every half hour. It is necessary, as would appear from the negative evidence furnished by several trials, that the powders should be given immediately succeeding the withdrawal of blood, lest, otherwise, the circulation recover its previous degree of excitement. The othe conditions of success are as follow: that "the treatment shall be enforced before the close of the fourth day, but earlier if possible; that the case shall be one of real acute rheumatism, not of the sub-acute or gouty form." This plan is quite similar to that pursued by Dr. Armstrong, who, however, used the pure opium, instead of the Dover's powder. It was the practice of Drs. Corrigan and Griffin to prescribe opium in one or two grain doses every second or third hour, "to be increased both as to quantity and frequency until there is decided relief, and kept at that dose, that is, the quantity to which it has been increased, until the complaint is steadily subsiding."

With respect to the comparative merits of opium and Dover's powder, I adopt the opinion expressed by Dr. Holland, in his Medical Notes and Reflections. Opium is almost always to be preferred. If we wish to combine ipecac with opium, it will be best to do so without the intervention of the sulphate of potassa, for that substance being inert, or, at any rate, of no explicable virtue, the quantity of Dover's powder required to produce the effects of a given dose of opium, say five grains, will constitute a mass too large and inconvenient for a single administration. Besides, whatever does no good may do harm.

3. *Inflammation of Serous Tissue.*—This article will be devoted exclusively to the elucidation of the opium treatment of peritonitis; for whatever is applicable to peritonitis may, *caeteris paribus*, be applied in the treatment of inflammation of the serous tissue in other parts of the body. It was my intention to have remarked briefly upon opium in pleurisy and arachnitis, but time and space forbid. It may be observed, however, that many distinguished practitioners, among whom I may reckon Prof. Flint, have shown, by their experience, that opium may be safely relied on in the treatment of the former; whilst it has been strongly recommended in the latter, by not a few of the most eminent of French physicians.

Having been made acquainted with the pathology of enteritis, regarding it, in a majority of instances, as an inflammation of the serous coat of the intestines, we should expect, by parity of reasoning, that the remedy most successful in its cure would be most appropriate in the treatment of peritonitis, which, as its name implies, is an inflammation of the lining of the abdominal cavity. Now, in enteritis, opium is our sheet anchor. Purgatives in both diseases are going out of fashion, to use a homely phrase, and opium is becoming more popular the more frequently it is properly used. The pamphlet put forth by Mr. Bates, of Sudbury, England, illustrative of the good effects of opium in peritonitis, seems to have made a strong impression on the minds of a few of the profession. Sometime *after* the publication of this pamphlet, I believe, Dr. Stokes related several cases of peritonitis from perforation, treated by Dr. Graves, with marked benefit, in some cases, in some again with absolute success. It was remarked of the condition of some of these patients, that the powers of life were so feeble that blood could not be abstracted, nor could mercury be trusted, throngh fear of exciting the peristaltic movements of the intestines. Opium seemed to be the only remedy i-dicated, and happily did it justify his anticipations. Is it not strange that physicians of the present day deny that opium can be relied on in the treatment of inflammation? If it be capable of relieving one of the most fearful and terrible maladies which has befallen the human race — peritonitis from perforation — is it unsafe to assume that the simple and uncomplicated variety may yield to its virtues?

Dr. Armstrong placed great confidence in opium, although he combined it with calomel, and drew blood previous to their administration. Such was the practice of our distinguished fellow-countryman, Dr. Post, long before the publication of Dr. Armstrong's works. The unvarnished narration, by Dr. Watson, of the case of Middlehurst, the tailor, is deserving the most serious attention. He had sporadic peritonitis. Blood-letting and calomel had both been vigorously employed, but without any appreciable good effects. So soon, however, as five grains of opium, in combination with calomel, were taken, the patient fell asleep, and "slept during the greater part of the night. Next morning his countenance had lost, in a great degree, its expression of anxiety; his belly was less tender, but still tense; and his tongue clean." Under the opium treatment the patient eventually recovered. And what is most conclusive, he grew worse whenever the opium was for a time withheld. With this case before us, we cannot avoid ascribing to opium an equal, if not the greater, share in the cure.

Dr. Churchill says, speaking of the use of opium in combination with calomel, "that the benefit of the opium in this combination, is not confined to the prevention of intestinal disturbance, but (that) it exerts a positive and beneficial influence upon the inflammation." "When the calomel acts on the bowels it may be omitted, and the opium alone continued; and I have seen as much benefit from it alone as from the calomel. Some years ago I saw a case of puerperul peritonitis, in consultation with a friend, and we administered large doses of opium (one grain every hour), with the greatest benefit. Since then, several similar cases have occured to me (Midwifery, p. 488, 1851, edition by Condie). Omitting the

3

vast accumulations of testimony which might be introduced in proof of the efficacy of opium in peritonitis, I conclude this already lengthened essay with a summary of the minutes which I noted from a lecture delivered in Bellevue Hospital, New York, February, 1852, by Prof. Alonzo Clark.

*First Case.*—First day—Announced by a chill; pulse 120, skin hot, tongue clean, respiration thoracic, abdomen tympanitic. Dr. Smith, of Bellevue, gave, according to Dr. C.'s instructions, ten minims of Magendie's solution of the sulphate of morphia.

2d day—Pulse 120, skin still hot, tongue not quite so clean, respiration thoracic, abdomen more tympanitic, vomiting of a greenish stuff like bile. One grain sulph. morphia every hour. Pulse slightly reduced.

3d day—Symptoms of second day still continuing; 1⅓ grs. morphia every hour. Slight narcotism induced; pulse fell to 90, the respiration to 7; vomiting continued, but the greenish color disappeared so soon as narcotism came on; 3 grs. of opium are now ordered every hour. The effects were, deeper narcotism, disappearance of the tympanitis and tenderness, fall of the respiration to 5; vomiting still persistent.

4th day—Diminished doses of opium; one grain every two hours, continued for four days. Surface moist, tongue slightly furred, no tenderness, no tympanitis. After the eighth day a dose of oil was given. The lochia were checked before the attack; at its commencement the milk secretion. For the first four days the patient averaged four grains of opium every hour.

*Second Case.*—Forty minims of solution of sulph. morphia was given the first hour; ½ drachm every hour until the fifth; sixth hour, 20 minims of the solution; from the sixth hour gradually increased to 24 grs. of opium; thus, in the following ratio, 6 grs., 7, 9, 11, 12, 16, 20, 24.

The dose was then diminished to 12 grs. The reasons for this were, that the respiration was only ten, the pulse reduced from 160 to 115, breathing became stertorous, and the mind wandering. So soon as the patient recovered from her mental aberration, 6 grs. of opium were administered. The symptoms increasing in severity during the second day, the quantity of morphia given was gradually carried to three drachms of Magendie's solution, equal to 30 grains of opium, having been preceded for three previous hours by 2½ drachms, or 25 grs., of opium. After this there was considerable vascular excitement; the pulse returned to 160, but, according to Dr. Clark, this effect was not due to the opium, for *it* has no tendency either to produce or to increase vascular excitement. It was remarked that when the skin was dry the patient became delirious, but when the surface became moist consciousness and the senses returned. Lactucarium was then substituted for opium, but in a short time the latter was again resumed, and continued in short doses until the sixth day, when all the symptoms of the disease passed away. On the seventh the patient had a spontaneous alvine evacuation, and the secretion from the uterus returned. The milk secretion did not appear until some time afterwards.

These were both cases of puerperal peritonitis; the second inordinately severe and unfavorable. Both were treated by opium alone; both terminated well. It is to be regretted that this report is not more ample and correct, for, as presented in these pages, it conveys but an imperfect and partial account of the line of treatment pursued by that distinguished physician. Let my apology for this report be found in an earnest desire to advance what he has convinced the author to be the proper method of managing this formidable complaint, a desire which I could never so fully realise otherwise than by transcribing to paper that which he so eloquently presented to his class by oral dissertation. Prof. Clark has been more successful in the treatment of peritonitis than any physician whose experience is on record. At the time he delivered the lectures to which I have referred, he had successfully managed eight cases out of nine of sporadic peritonitis. From the 10th October, 1851, to the 21st February, 1852, eleven cases of puerperal peritonitis had occurred in Bellevue. Three, who died, were not seen until the third day of the disease, and should not, therefore, be reckoned among those who secured *full* treatment. Of the remaining eight who were visited on the first day, two died, but both of them had inflammation of the uterus conjoined with the peritonitis. Six recovered. We may then say that all the cases of true peritonitis that were seen on the first day terminated favorably. Whether allowances be made for the lateness of the hour at which the three first cases were seen, or for the inflammation of the uterus, which occurred in two, it must be admitted that the success which Dr. Clark has obtained by the opium treatment is greater than has ever attended any other mode of practice.

I shall merely mention, without going into the particulars, a most violent and unpromising case of traumatic peritonitis, supervening in consequence of a gun-shot wound in the abdomen, which was successfully treated by Dr. W. Cooke, formerly of Nicholasville, Ky., with large quantities, oft repeated, of the tinctura opii. The patient was robust and plethoric, addicted to intemperance, and, at the time of the reception of the injury, was in a state of intoxication. The same physician succeeded in restoring to health, by like means, a negro woman, afflicted with puerperal peritonitis, who was, in the opinion of an intelligent medical gentleman present, on the very verge of the grave. The author may be excused for alluding to these two cases, on account of the scarcity of evidence which we possess of the virtues of opium alone in the treatment of such diseases.

*Modes of Administration.*—They may be arranged under four distinct heads, according to the object to be effected, and to the condition of the system at the time of administration.

1. *Opium alone.*—It has frequently been observed in these pages that irritation is the first and chief element of inflammation ; or, as Prof. Rogers has remarked, it is the first link in the chain of phenomena constituting this morbid affection. By quieting this irritation opium removes, so to say, the prop of the disease, it nips the inflammation in its budding. This is the mode, and this the stage, in which opium especially manifests its virtues in controlling inflammation.

2. *Opium after Venesection.*—After the inflammation has become fully developed, irritation having been succeeded by morbid changes in the vascular system, we cannot, with so much certainty or confidence, rely upon opium exclusively. The lancet may be called in to subdue the inflammation, and opium administered afterwards to prevent its rekindling. The lancet produces sedation of the system, which is maintained by the opium. In both this and the preceding case the opium must be given in large doses.

3. When the lancet cannot be used on account of prostration, feebleness of the system, etc., opium may be given to sustain the flagging powers until the morbid action has passed off or exhausted its venom. This practice is especially recommended in inflammations arising after hemorrhages, in the course of debilitating fevers, and in such inflammations of the abdomen as require perfect repose of its contents.

4. *Opium in combination.*—In this way it is applicable to all stages of acute inflammation. The articles with which it is usually associated are, tartrate of antimony and potassa, mercury and ipecacuanha. Thus combined it is generally administered in moderate doses. It is supposed that the ipecacuanha, the mercury, etc., counteract the stimulating effects of the opium, whilst the latter the more readily insure the constitutional effects of its associated remedies.

